# The influence of bank governance structure on green credit

**DOI:** 10.1371/journal.pone.0281115

**Published:** 2023-03-13

**Authors:** Fengge Yao, Zenan Qin, Xiaomei Wang

**Affiliations:** 1 School of Finance, Harbin University of Commerce, Harbin, Heilongjiang, PR China; 2 School of Tourism and Culinary Arts, Harbin University of Commerce, Harbin, Heilongjiang, PR China; University of Almeria: Universidad de Almeria, SPAIN

## Abstract

The introduction of green credit policy provides an important idea to solve the contradiction between economic development and environmental protection. Based on fuzzy-set Qualitative Comparative Analysis (fsQCA) method, from the perspective of bank governance structure, this paper selects ownership concentration, independence of the Board, executive incentive, activity of Supervisory Board, degree of market competition and loan quality as antecedent variables to analyze the path of their impact on green credit. It is found that: (1) The main way to achieve high-level green credit is high ownership concentration and good loan quality. (2) The configuration of green credit has causal asymmetry. (3) Ownership structure is the most important factor affecting green credit. (4) There is a substitution between the low independence of the Board and the low executive incentive. The low activity of Supervisory Board and the poor loan quality are also substitutable to a certain extent. The research conclusion of this paper is helpful to improve the green credit level of Chinese banks and win the green reputation for banks.

## 1. Introduction

In recent years, China’s economy has maintained rapid growth, and its total economic output has jumped to the second in the world. The rapid economic development has also brought a series of problems such as resource depletion, environmental pollution and ecological deterioration [1, 2]. Green credit, as a new financing mode, alleviates the contradiction between economic development and environmental protection [3, 4]. As the main executor of green credit policy, banks play an important role in national economic development [5, 6]. According to statistics, by the end of 2020, the green credit balance in China’s banking industry had reached 7906.5 billion yuan, a year-on-year increase of 17.73%. In fact, the total amount of green credit continues to expand, but the proportion of green credit balance in total loans is still low, and its contribution to economic growth is small, which cannot effectively meet the needs of green economic development. Therefore, in the context of green development, whether improving the bank governance structure will help to improve the level of bank green credit for bank? Based on the above considerations, this paper studies the effect of bank governance structure on green credit from the perspective of internal and external governance structure of banks, so as to obtain the way for banks to improve the level of green credit.

Foreign scholars have studied green credit earlier. During the research, Thompson and Cowton (2004) found that whether banks grant loans to enterprises largely depends on the impact of the project on the environment. Banks will make decisions according to its impact. Therefore, they call this process “Green Credit” [7]. Narayan and Sharma (2015) discussed the green measures taken by contemporary global banks from the perspective of carbon emissions and found that banks in North America and Europe are the pioneers in adopting green credit practices [8]. Huyghebaert (2006) believed that the green reputation obtained by enterprises is the comprehensive result of all friendly behaviors for environment in the past, and the active implementation of green credit policy is the main way to implement friendly behaviors for environment [9]. Mori et al. (2013) studied Citibank and ABN AMRO, and they analyzed the successful practical experience of bank green credit in several countries such as the United States, the United Kingdom and Japan, and proposed that the government should establish policy supervision mechanism and improve the guarantee system and legal system to make green credit benefit more stakeholders [10]. Vander-Walt et al. (2014) started with green credit, and put forward that the bank’s green credit standards need to be unified and the corresponding standards need to be specific [11]. They also put forward constructive suggestions that all departments of various countries should cooperate with each other to help the common development of the global economy, banks should improve their core competitiveness and improve the risk management system. Haque and Shahid (2016) found that the impact of bank ownership structure on credit risk shows the fine governance of the banking industry by using 390 observation samples of 32 commercial banks from 2000 to 2014, and strongly recommended that banks should be denationalized or reduce government ownership [12]. Some scholars believe that the implementation of green credit is conducive to the development of banks. Cilliers (2012) studied the impact of bank green credit business on its sustainable development. Through a series of studies, it is concluded that there is a positive correlation between bank green credit business and bank sustainable development [13]. On the basis of previous studies, Richardson (2014) used theoretical research methods to prove that green credit cannot only greatly reduce the operating costs, but also help banks to carry out other businesses for a long time and effectively [14]. Others believe that the implementation of green credit is not conducive to the development of banks. Wright (2012) considered that there are differences in the capital needs of enterprises [15]. The market’s own adjustment mechanism can effectively promote the flow of credit resources to appropriate industries. Therefore, the implementation of green credit will only increase the cost of banks.

China’s green credit was born in 2007. It has only been implemented for more than ten years, far behind foreign developed countries. At present, it is still in the primary development stage. Zhu and Yu (2011) used game theory to analyze the interest relationship among banks, enterprises and government in the implementation of green credit policy, and found that the Nash equilibrium between the government and enterprises is related to the fines of polluting enterprises and the supervision and inspection costs [16]. Hu et al. (2011) believed that the green credit policy is an important measure to achieve the goal of energy conservation and emission reduction and turn to the development of green economy [17]. After the implementation of the policy, the credit structure of China’s commercial banks has changed, but there are still some deficiencies in the policy, such as the lack of unified credit standards, insufficient information disclosure and so on. Li et al. (2012) pointed out that since the implementation of the green credit policy in July 2007, commercial banks have increasingly supported energy-saving measures, but the loan scale of high energy consuming companies is still increasing [18]. There are few studies on green credit in China. Most studies still focus on the concept, development status and action mechanism of various factors to explain the related problems of bank performance, risk and so on. It is obvious that existing researches does not consider social and economic development and it is lack of connection with practical operation.

As a supplier of funds through credit operation, banks need to invest more funds in low emission and environmental protection enterprises, which plays a vital and even decisive role in green credit projects. Good bank governance structure can reduce credit risk and improve credit quality. Bank governance is an important direction of corporate governance, and their main structures are consistent. Ciancanelli and Reyes-Gonzalez (2000) put forward the basic theoretical system of bank governance [19]. By studying six emerging markets, Newell and Wilson (2002) found that the best corporate governance level will bring 10%-12% premium return effect to enterprises compared with the worst corporate governance level [20]. Klapper and Love (2004) pointed out that corporate governance can bring positive benefits to enterprises [21]. Gompers et al. (2003) used American IRRC corporate governance data to construct a governance index, which confirmed that there is a significant positive correlation between corporate governance index and stock return [22]. In the past, most of the research on corporate governance used traditional qualitative or quantitative research methods. It is obvious that the qualitative research method has weakness of lacking universal applicability and wide popularization [23, 24], this is because qualitative research methods need to summarize the rules of single case or conduct logical reasoning in application [25]. The quantitative research method has achieved universal applicability, but it also has its own shortcomings [26, 27]. The basic assumption of quantitative research method is that independent variables play an independent role and focus on the atomic perspective. However, the social phenomenon is generally complex and affected by many factors. Under the thinking of quantitative research methods, it is difficult to establish a global view and a holistic view to solve problems. Therefore, it has a defect which cannot solve the problem of causal complexity [28]. Qualitative Comparative Analysis (QCA) can not only overcome the general applicability of qualitative research methods, but also break through the obstacles that quantitative research methods cannot take into account the whole. In conclusion, compared with traditional analysis methods, QCA has the following characteristics: Firstly, QCA abstracts the object to be studied into different factor combinations, and uses the idea of set theory to judge the subordinate relationship of the set to which the factors belong. Secondly, the subordination under the idea of set theory is asymmetric, which means a result can correspond to multiple paths, therefore this method implies multiple concurrent causality. Finally, it is an upgraded version of ordinary qualitative research. Although it pays more attention to qualitative change, it adds scientific statistical verification to qualitative analysis.

There are few literatures on studying bank governance by using QCA, most studies focus on discussing corporate governance. The four main theories of corporate governance include: two rights separation theory, principal-agent theory, stakeholder theory and capital structure theory. Most scholars start from listed companies, but different types of companies have different governance models. Jackson (2005) conducted an empirical analysis on how corporate governance, trade union power and political system of 22 countries and organization samples lead to the joint decision-making of the Board [29]. Garcia and Casasola (2011) studied 6611 family businesses in 46 countries and obtained various configurations composed of ownership structure, board governance, management governance and inheritance, and they classified family businesses according to different governance methods [30]. By studying the governance of 198 companies in 36 countries with Initial Public Offerings (IPO) in the United States, Bell et al. (2014) concluded that different incentives and combinations of incentive governance methods can bring equal value to investors [31]. Legault-Tremblay (2015) analyzed the interaction between the governance conditions of 1337 listed companies in China, and drew relevant conclusions on corporate governance with Chinese characteristics [32]. It can be seen that the governance modes of different companies in different countries are quite different. Whether IPO companies with capital characteristic or traditional family enterprises, they have unique governance modes, and the factor combination needs to be adjusted according to the actual situation. In the process of studying corporate governance by using QCA method, different researchers choose different indicators. Felicio et al. (2016) studied the governance structure of 32 commercial banks in the UK and adopted the “non-performing asset ratio” to measure the loan quality [33]. At present, there is no direct empirical study on the relationship between bank governance structure and green credit. From the perspective of bank governance structure, through the combination of theoretical analysis and empirical analysis, this paper selects fsQCA method, extracts and condenses six antecedent factors (ownership concentration, independence of the Board, executive incentive, activity of Supervisory Board, degree of market competition and loan quality) to explore how Chinese banks can achieve a high level of green credit through the combination of these factors.

## 2. Theoretical framework and case selection

### 2.1 Ownership structure

Ownership structure determines the relationship between bank owners and their entrusted agents, which is the core content of bank governance structure. Berle and Means (1932) first studied the ownership structure and believed that the ownership represents the control of the company [34]. The ownership structure refers to the composition of the company’s ownership, that is, who is the owner of the company. Its content involves the shareholding ratio and ownership distribution of shareholders, which are closely related to the management and operation of banks. According to the actual development of the bank, reasonably arranging the ownership structure is of great significance for the bank to improve green credit and achieve rapid development. Shleife and Vishny (1989) emphasized the protection of property rights and stakeholders in relevant laws from the perspective of governance globalization [35]. Using the data of 13 European banks from 1998 to 2004, Shrieves et al. (2010) found that the characteristics of “equity friendly” and “credit friendly” markets have a positive effect on bank capital [36]. The relative static characteristics of the stock and credit markets on which banks rely reduce the ongoing and potential integration of European banking markets.

Ownership concentration is an important indicator to measure the distribution status and stability of bank equity [37, 38]. The assets of a joint-stock bank are divided into equal shares, and each shareholder enjoys the corresponding income right and control right according to the proportion of capital contribution. The research on the distribution of equity is important because the concentration of equity determines different market behaviors of banks through the distribution of control rights, and has different effects on the role of bank governance mechanism [39], which will have an impact on green credit. Existing studies have always owned different views on the relationship between ownership concentration and corporate governance. Bolton and Thadden (1998) and Burkart et al. (2000) believed that the higher the ownership concentration, the more unfavorable it is for the management layer to make correct decisions, to enhance the value of the company, and will seriously hinder the development of the company [40, 41]. Pawlowska (2016) and Musah (2018) studied the balance sheets of 72 commercial banks in 10 Middle East and North Africa (MENA) countries from 2000 to 2016, and found that ownership concentration is positively correlated with bank risk, and foreign banks have greater risk than state-owned banks [42, 43]. However, Kapopoulos and Lazaretou (2007) believed that high ownership concentration has a positive impact on the development of the company [44]. Ismail and Sinnadurai (2012) studied the quality and ownership concentration of corporate governance, and made use of the performance of ISBEIA to conclude that the ownership concentration matching the enterprise’s own situation is beneficial to improve the quality of corporate governance [45]. Another scholar believed that there is no significant relationship between ownership concentration and bank development, but it will reduce loan risk [46, 47]. There are both contradictions and convergence of interests between controlling shareholders and other shareholders. Its consistency is that all shareholders are consistent in preventing insiders from encroaching on the interests of owners. They are also consistent in achieving the goal of maximizing market value, improving operation and management and maximizing earnings per share. Therefore, it is particularly important to study the impact of ownership concentration on green credit as one of the constituent elements of bank governance.

### 2.2 Board characteristics

As an important part of the bank governance structure, the Board characteristics are directly related to the bank’s decision-making and shareholders’ interests. As a link between shareholders and management layer, it is used to solve the agency problem between them [48]. As for the Board characteristics, most studies stress the Board size and independence. Lipton and Lorsch (1992) and Pathan et al. (2007) believed that the larger the Board size, the higher the agency cost of the bank in the process of governance, and the more likely it is to cause a waste of resources, which is very unfavorable to the bank’s operation and decision-making [49, 50]. Agnihotri and Bhattacharya (2017) empirically analyzed the panel data of listed manufacturing companies, and found that the increase of the Board size is not conducive to the company’s decision-making, and the relationship between the proportion of independent directors in the Board and performance is not significant [51]. However, Belkhir (2009) reached the opposite conclusion and found that there is a significant isotropic relationship between the Board size and Tobin Q [52]. By conducting empirical test, for some companies with high professional requirements, such as those involved in multiple industries, large-scale or highly leveraged companies, Coles et al. (2008) found that the larger the Board size, the higher the proportion of internal directors, and the more profitable the company is [53]. At present, the research conclusions are inconsistent on whether the large scale of the Board is conducive to the development of the company.

The independent director system is an important part of the Board characteristics. Independent directors can objectively evaluate the decisions of the Board. When the interests of the management layer and the company are inconsistent, independent directors can play a better role. A perfect independent director system is not only conducive to strengthening internal checks and balances and improving corporate governance, but also conducive to protecting the interests of shareholders. Therefore, countries all over the world began to implement the independent director system. Yermack (1996) and Andres et al. (2005) used mixed industry enterprises as samples to study the relationship between board independence and corporate value, and found that there is no significant relationship between them [54, 55]. Subsequently, Adams and Mehran (2003) confirmed this conclusion [56]. The empirical results of Agrawl and Knoeber (1998) showed that independent directors sometimes made misleading business strategic decisions [57]. Theoretically, the strong independence of the Board can weaken insider control and reduce agency costs, so as to promote the Board to play an active role in bank management and resource allocation and improve the governance efficiency of the Board. Andres and Vallelado (2008) conducted a global sampling analysis and believed that the higher the proportion of independent directors, the more conducive to the development of the company, and there is not a simple linear relationship between the Board size and bank performance, but a U-shaped relationship [58]. The research results of Kim (2010) showed that most independent directors had expertise in various fields such as legal affairs and subdivided industries, which can add some irreplaceable resources to the Board to make effective decisions [59]. Taking American public oil and gas companies as a sample, Post et al. (2015) believed that the higher the proportion of independent directors, the more likely the company is to form an alliance with sustainability as the theme [60]. Malik and Makhdoom (2016) selected global Fortune 500 enterprises for conducting research [61]. The results show that the existence of independent directors makes the process of strategic decision-making more transparent. The research results of Karim et al. (2020) showed that independent directors are the intermediary point between the external environment and the company [62]. Independent directors are more convenient to visit partners and customers in the industrial chain, collect more external information for the company, and make scientific and reasonable decisions. As for whether the high proportion of independent directors is conducive to the development of the company, the current research conclusions are not consistent. The research on the independence of the Board and green credit is zero.

### 2.3 Management status

How to optimize the manager’s behavior so as to make its operation and management behavior conducive to maximize the company’s interests as well as managers’ own interests is the focus of current research. Avoiding the loss caused by moral hazard is a typical research topic in Management. Therefore, incentive mechanism is applied under this background. As a mean to stimulate the management to work hard, executive incentive plays an increasing role in the company’s business process [63]. Anderson and Fraser (2000) studied the Japanese banking industry from 1977 to 1996 and found that banks did not give sufficient incentives to senior executives, resulting in inefficient bank governance structure, which may lead to the crisis of Japanese banking industry [64]. Executive incentive is divided into equity incentive and salary incentive. Mehran (1995) and Kato et al. (2007) explored the relationship between executive equity incentive and company performance with mixed industry enterprises as samples, and they found that there is a significant positive correlation between them [65, 66]. Ahmad et al. (2012) pointed out that there is a significant positive correlation between executive shareholding ratio and corporate governance, indicating that executive equity incentive plays an obvious role in improving governance level and performance [67]. Executive compensation can link the interests of the management layer and shareholders, so as to improve the enthusiasm and effort of the management layer and promote the efficiency of resource allocation. Cui and Mak (2002) found that executive compensation can promote the development of the company [68]. Cornett et al. (2009) took large American banks as research samples and found that under the condition that executive compensation is linked to performance, there is a significant positive correlation between them [69]. However, Evans and Evans (2002) believed that they are not related. At present, there is little research on executive compensation and green credit [70].

### 2.4 Governance of Supervisory Board

The Supervisory Board is an organization, elected by the general meeting of shareholders, to exercise supervision functions. The supervision objects are mainly the Board and Managers. Due to the different political system and economic system between China and Western countries, the function of bank governance has not been brought into full play and displayed, among which the lag of the governance of Supervisory Board is more serious [71]. Therefore, in the practical practice of bank governance in China, the Supervisory Board is known as “Scarecrow”.

The research literature on the governance structure of Supervisory Board is relatively few, because the Supervisory Board, as an internal organization to supervise the Board and Managers to correctly exercise their functions and powers, usually does not exercise their functions and powers alone as a bank governance organization. Although the governance of Supervisory Board has been weaker than that of the Board of directors in terms of strength and efficiency, it has attracted the attention of bank governance researchers, because the governance of Supervisory Board, no matter in aspect of theory or practice, is an indispensable element in the bank governance system [72, 73]. Marcinkowska (2012) believed that the impact of supervision should be considered in bank governance [74]. In the empirical study, Hu et al. (2010) found that most supervisors in China are highly consistent with the interests of major shareholders, which is naturally not conducive to the development of the company [75].

The Supervisory Board has the characteristics of independence, legality and professionalism. The more active of Supervisory Board is, the more it can strengthen the supervision of the bank’s internal management and operation. If the Supervisory Board finds that the directors and senior executives have committed acts against the interests of the company, it may require them to stop, and has the right to organize and convene an extraordinary general meeting of shareholders if necessary. An effective Supervisory Board can represent the interests of the majority of shareholders, actively urge the bank’s management layer to establish a perfect green credit system, and reduce the possibility that managers use information asymmetry to damage the interests of shareholders and banks. Theoretically, the more active of Supervisory Board is, the more conducive it is to the realization of this supervisory role and to the improvement of the level of green credit. However, the implementation of green credit policy is also affected by other factors.

### 2.5 Degree of market competition

External governance and internal governance are inseparable [76]. Some scholars believe that internal governance and external governance are alternative relationships. Williamson (1983) put forward the “alternative interpretation” and believed that if the external governance structure is weak, the effect of internal governance will be more obvious [77]. This hypothesis has been empirically supported by some scholars. Tian and Twite (2011) found that external governance structure (such as product market competition) will affect internal governance [78]. When the company is in a highly competitive environment, the effectiveness of internal governance is weakened, supporting the “alternative interpretation”. Chou (2013) reached the same conclusion. Other scholars believe that internal governance and external governance are complementary [79]. Grosfeld and Tressel (2001) studied that both product market competition and owner structure have a significant impact on the company’s productivity growth [80]. After conducting further research, they also found that product market competition and owner structure will promote each other rather than replace each other.

The role of market competition on corporate governance is mainly reflected in three aspects: corporate control, manager market and product market. In China, bank mergers and acquisitions spontaneously formed in the market are very rare, so the control competition is difficult to become an effective bank governance structure. In addition, due to the consideration of financial security, the appointment and removal of bank managers in China need to be reported to the Banking and Insurance Regulatory Commission for review. The market competition of bank managers has received a high degree of government intervention, and there is no market-oriented manager system. With the development of financial liberalization and financial innovation, the competition in the banking industry is increasing. The competition for market share among banks is becoming increasingly fierce, the degree of product homogeneity is gradually improving, and the product market competition has an increasing impact on bank governance. At present, theorists generally agree that product market competition can promote the development of the company. For example, Nickell et al. (1997) and Januszewski et al. (2002) showed that the more competitive the product market is, the faster the company’s productivity increases [81, 82]. Arun and Turner (2009) studied the banking governance of developing countries and found that developing countries should promote their banking governance by improving the degree of product competition [83]. Furthermore, improving the level of bank governance can promote banks to actively respond to the national call for “green finance” and improve the level of green credit. At present, there is no direct study on the impact of market competition on green credit.

### 2.6 Loan quality

Loans are the main assets of banks. In the loan transaction with the borrower, the bank will formulate different transaction conditions and conduct one-to-one contract transaction according to the loan application made by the borrower with different credit levels. As the loan is a non-market transaction, it is generally not quoted and transferred in the open market, which makes the loan quality difficult to observe and increases the degree of information asymmetry. In this case, in order to protect the interests of stakeholders, bank governance needs to supervise the loan quality and strictly control the loan risk. Through the supervision of loan quality, it can exert pressure on banks to force them to fulfill their duties in the process of conducting loan transactions, control loan risks and improve the level of green credit.

**Fig 1:**
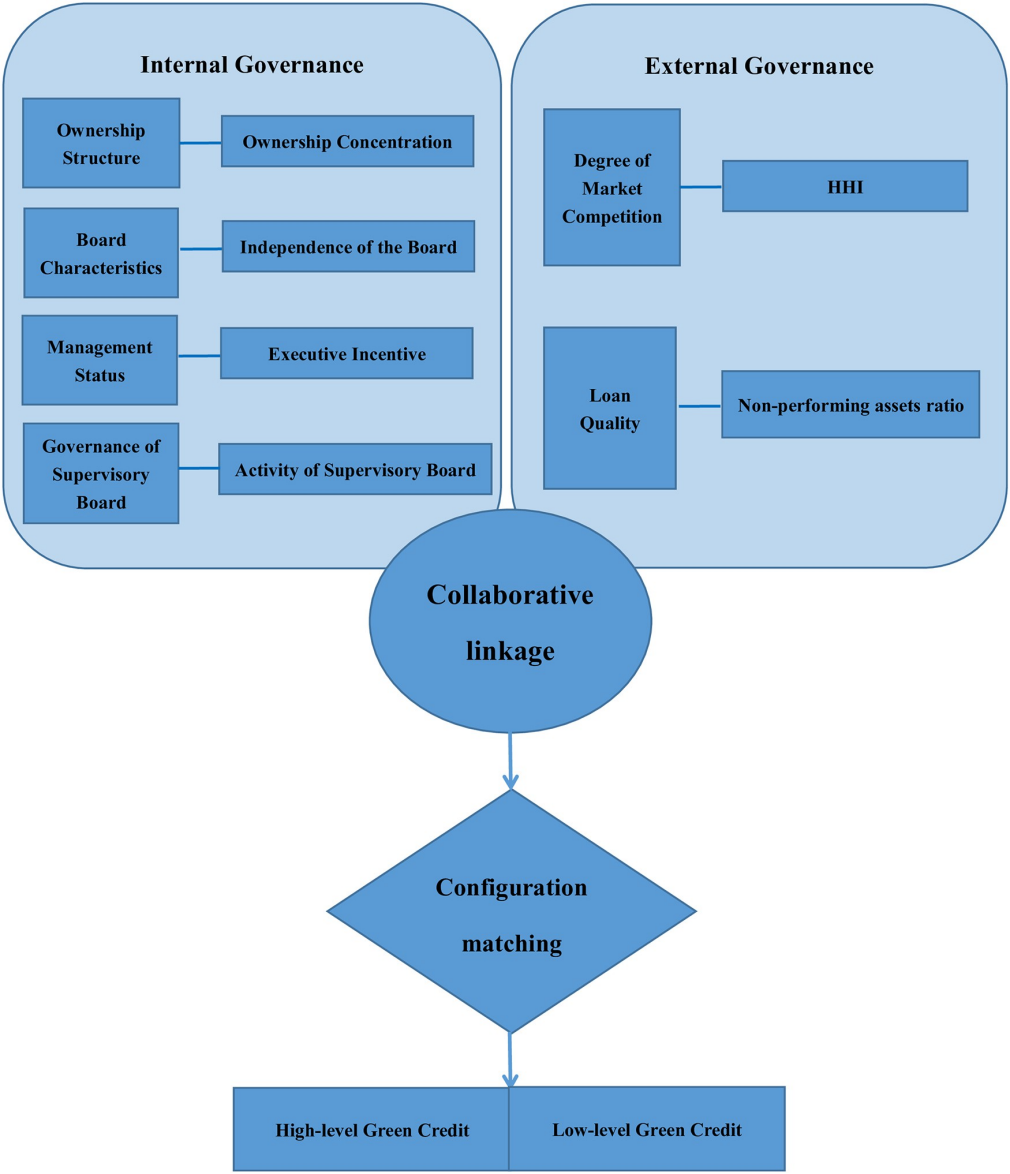
Theoretical framework of the influence of bank governance structure on green credit.

Generally speaking, the research on the impact of bank governance structure on green credit by domestic and foreign scholars is almost zero. Based on theory and literature, this paper summarizes the identified conditional factors into six aspects: ownership structure (ownership concentration), Board characteristics (independence of the Board), management status (executive incentive), governance of Supervisory Board (activity of Supervisory Board), degree of market competition and loan quality. Moreover, the ownership structure, the Board Characteristics, the management status and the governance of Supervisory Board belong to internal governance. The degree of market competition and loan quality belong to external governance. Accordingly, this paper develops a configuration framework and establishes a configuration model based on it, as shown in Fig 1.

### 2.7 Case selection

In the screening process of the specific sample banks, after excluding the banks with ST and *ST, banks that issues B shares and banks with missing variable values, the remaining 12 banks are the sample banks of this paper. They are: China Development Bank, Bank of China, China Construction Bank, Industrial and Commercial Bank of China, Agricultural Bank of China, Guangdong Development Bank, Shanghai Pudong Development Bank, China Merchants Bank, China CITIC Bank, China Everbright Bank, China Huaxia Bank and China Bohai bank. Among them, China Development Bank, Guangdong Development Bank and China Bohai bank are non-listed banks. The data of these 12 banks from 2014 to 2019 are selected for research.

## 3. Study design

### 3.1 Overview of Qualitative Comparative Analysis

QCA method is a “case oriented” method to solve the interdependence and causal complexity of configuration phenomena, which was proposed by Ragin in 1987 [84–86]. In 2007, Fiss analyzed QCA and its possible combination with strategic research, and opened the application of QCA in organizational management research fields such as strategy [87]. fsQCA is to further calibrate the factors as set membership score on the basis of qualitative dichotomy. The set membership score is not limited to 0 (no membership) or 1 (full membership). Therefore, the basic idea of fsQCA is to allow the scaling of set score, that is, to allow partial membership.

For this paper, fsQCA is an appropriate analysis method for the following reasons: Firstly, previous studies have shown that bank green credit is a complex phenomenon caused by the interaction of internal and external governance structures. Therefore, to explore the paths to improve bank green credit, only the statistical analysis based on the independent effect or pairwise interaction between antecedent variables is not enough to explain how different conditions affect green credit. fsQCA method starts from holism and analyzes the complex causality among many factors, which just makes up for this defect. Secondly, there are many equivalent causal chains to enhance green credit. fsQCA method is helpful to identify the antecedent configuration with equifinality results, understand the differentiation mode leading to the improvement of green credit under different antecedent conditions, and further discusses the complementary and alternative relationship between conditions. Thirdly, from the perspective of management practice, even if some banks actively respond to the green credit policy, it is still difficult to avoid the low level of green credit. fsQCA can better answer the asymmetry problem, that is, the paths for high-level green credit and low-level green credit are not opposite to each other. Fourth, this paper selects the data of 12 banks from 2014 to 2019 as case samples for conducting research, and it investigates many influencing factors of their internal and external governance structure, which is difficult to obtain stable results through statistical methods. fsQCA method is not only suitable for cross case studies of small, medium and large samples, but also can conduct analysis from multi angle, which ensures the external popularization of the results of this paper to a certain extent.

fsQCA believes that there are different combinations of reasons for the occurrence of the same social phenomenon. These combinations of reasons can be regarded as possible set ideal paths. Then, through the control of the two important parameters of consistency and coverage, the most explanatory logical condition combination can be judged, and finally the theoretical explanation of the phenomenon can be obtained. The calculation formula of consistency and coverage is as follows:

$$\text{consistency}\left(A\leq B\right)=\frac{\sum \text{min}\left(a_{i},b_{i}\right)}{\sum a_{i}}$$ (1)

$$\text{coverage}\left(A\leq B\right)=\frac{\sum \text{min}\left(a_{i},b_{i}\right)}{\sum b_{i}}$$ (2)

Where *a*_*i*_ represents the membership degree of individual i in combination A and *b*_*i*_ represents the membership degree of individual i in result B. The range of Consistency is 0 to 1. When the value is 1, it indicates that A completely belongs to B. That is, the stronger the adequacy. If it is higher than the acceptable minimum empirical standard, it is meaningful to carry out the next set analysis. Coverage can be interpreted as the extent to which the set relationship through the consistency test explains the results. The subset relation of a set expresses a kind of “sufficient and unnecessary” relation. Its range is 0 to 1. When the value is 1, it shows that the set has the strongest explanatory power to the result, that is, the combination of factor A is the only way to achieve the result of B.

**Fig 2:**
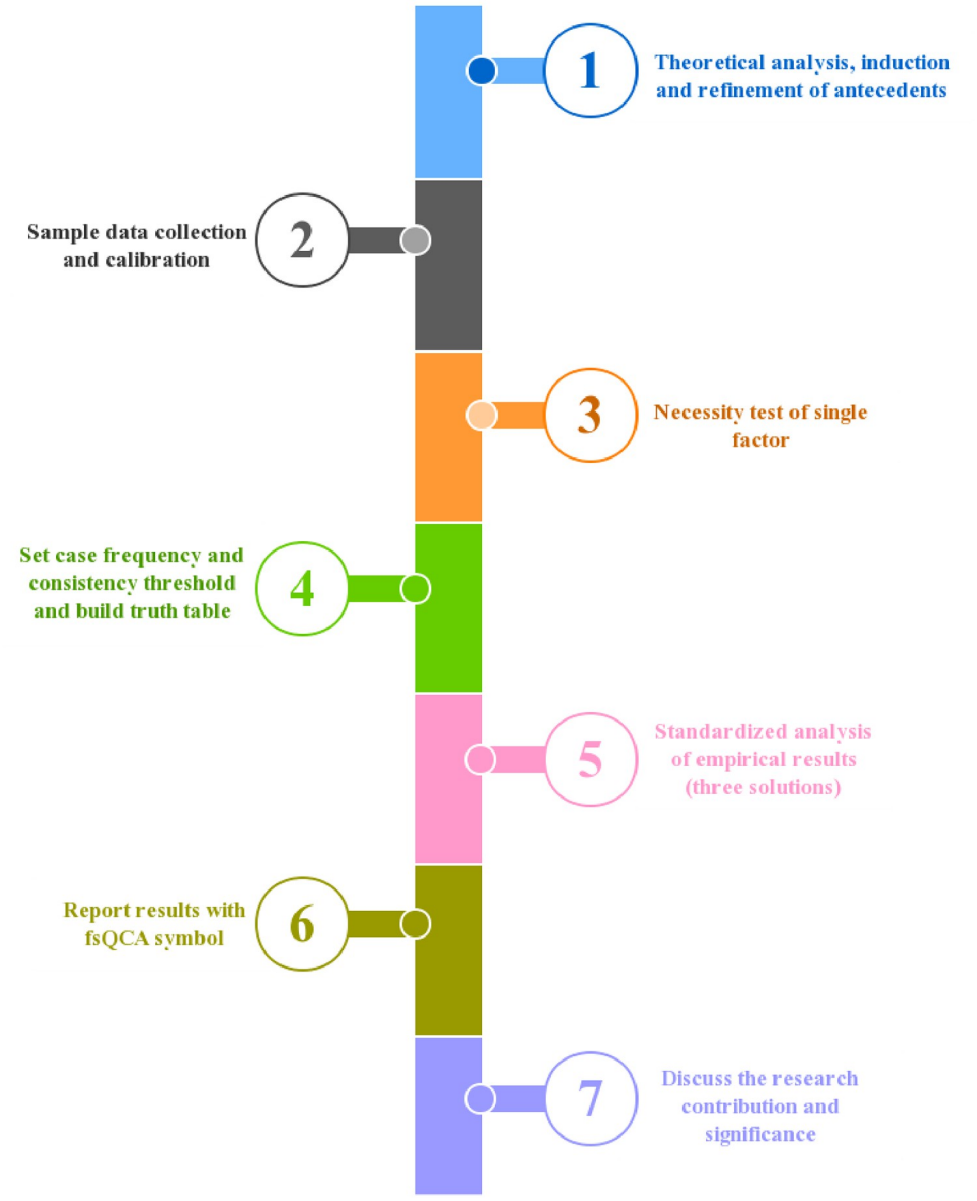
Research steps under fsQCA method.

As shown in Fig 2, the research steps are generally “calibration variables- variable calibration- condition test- truth table calculation- path analysis”.

### 3.2 Variable selection

In fsQCA method, the selection of variables is very important. With the increase of antecedent variables, the number of multiple conditional concurrency causes will increase logarithmically (2^n^). Generally speaking, when the sample cases belong to medium samples, the optimal number of antecedent variables is 3–8. This paper selects 12 banks from 2014 to 2019 as research cases. In order to prevent the emergence of limited diversity, this paper can select a maximum number of 6 antecedent variables. Specific variables are defined and explained as follows.

#### 3.2.1 Antecedent variables

*3.2.1.1 Ownership structure*. The variable of ownership structure is expressed by ownership concentration. Abbreviate it as OC. The shareholding ratio of controlling shareholders is an important index to measure the ownership structure of banks. It not only affects the stability of ownership structure, but also affects the decision-making efficiency and scientificity of banks. Ownership concentration is measured by the shareholding ratio of the first largest shareholder. The calculation formula is as follows:

$$SRFS=\frac{SFS}{TB}$$ (3)

Where SRFS represents the shareholding ratio of the first largest shareholder, SFS represents the number of shares held by the first shareholder, and TB is the total number of shares of the bank.

*3.2.1.2 Board characteristics*. The variable of Board characteristics is expressed by independence of the Board. Abbreviate it as IB. Independent directors refer to those who have no personal and economic interests with the bank’s management layer and can independently supervise the behavior of the management layer. Independent directors should have at least two characteristics: Firstly, they are more impartial than directors with special interests. Secondly, they own decision-making knowledge. A bank that performs well in terms of independent directors can reflect the effectiveness of internal governance structure to a certain extent and enhance the confidence of investors, thus playing positive role in producing green credit. This paper selects the proportion of independent directors to measure the independence of the Board, and then explores the impact of the Board characteristics on bank green credit. The calculation formula is as follows:

$$PID=\frac{ID}{TB}$$ (4)

Where PID represents the proportion of independent directors, ID represents the number of independent directors, and TB is the total number of Board.

*3.2.1.3 Management status*. The variable of management status is expressed by executive incentive. Abbreviate it as EI. Compensation incentive is a very important factor in executive incentive, and it is the most basic and extensive incentive method. Compensation can affect the diligence and effort of bank management to a certain extent. This paper selects executive compensation incentive to measure the situation of management. Because the amount of compensation is too large, this paper uses the natural logarithm of the total annual salary of executives as the measurement index. That is *EI* = *LN*(*total annual salary of senior executives*).

*3.2.1.4 Governance of Supervisory Board*. The variable of governance of Supervisory Board is expressed by the activity of Supervisory Board. Abbreviate it as ASB. The meetings of Supervisory Board provide a place for communication among members. The number of meetings of Supervisory Board is a characteristic of the diligence of the members of Supervisory Board. The more meetings of Supervisory Board, the more active the supervisors will be in performing their supervisory duties. The meeting of Supervisory Board can improve the transparency of bank management and decision-making. Based on the supervision subject and authority of China’s Supervisory Board, regular meetings of board of supervisors indicate that the Supervisory Board has a sense of responsibility and can spend more time and energy to conduct its supervision duties, so as to make the bank governance structure more reasonable. The activity of Supervisory Board is expressed by the average number of meetings of Supervisory Board held each year.

*3.2.1.5 Degree of market competition*. The variable of degree of market competition is expressed by Herfindahl Hirschman Index (HHI), and it is measured by product market concentration. HHI is a reverse indicator. The larger the HHI, the lower the product market competition. The closer HHI is to 0, the more competitive the product market is. The calculation formula of HHI is as follows:

$$HHI={\sum \left(\frac{BOI}{IOI}\right)}^{2}$$ (5)

Where BOI represents the bank operating income, and IOI is the total industry operating income.

*3.2.1.6 Loan quality*. The variable of loan quality is expressed by non-performing asset ratio. Abbreviate it as LQ. The calculation formula is as follows:

$$NPA=\frac{WOBD}{TL}$$ (6)

Where NPA represents the non-performing asset ratio, WOBD represents the write off of bad debts, and TL is the total loans.

Bad debts refer to loans that have been overdue or extended for more than 3 years and have not been recovered. Write off of bad debts is the bank’s behavior of confirming the loss of assets that meet the recognition conditions of bad debts. After writing off the bad debts, it will stop accounting in the balance sheet and put into off balance sheet management. Therefore, write off of bad debts can be understood as recognition of bad debts. Therefore, the higher the proportion of bad debts written off, the worse the loan quality. That is, the higher the non-performing asset ratio, the worse the loan quality.

#### 3.2.2 Outcome variable

The variable of green credit is abbreviated as GC and measured by the logarithm of green credit balance. That is, *GC* = *LN*(*green credit balance*). The green credit balance represents a bank’s implementation effect of the green credit policy. The higher the green credit balance, the higher execution strength of the green credit policy. The specific explanation of variables is shown in Table 1.

**Table 1:** The specific explanation of variables.

Category	Variables	Code	Meaning and description
Ownership Structure	Ownership Concentration	OC	Shareholding ratio of the first largest shareholder
Board Characteristics	Independence of the Board	IB	Number of independent directors/total number of Board
Management Status	Executive Incentive	EI	Ln(total annual salary of executives)
Governance of Supervisory Board	Activity of Supervisory Board	ASB	Meetings of Supervisory Board
Degree of Market Competition	HHI Index	HHI	∑(Bank operating income/industry operating income)^2^
Loan Quality	Non-performing assets ratio	LQ	Write off of bad debts/total loans
Green Credit	Green Credit Balance	GC	Ln(green credit balance)

### 3.3 Data sources

The sample data of this paper mainly comes from the public information of listed banks, including the company’s annual report, company announcement, articles of association, Wind financial terminal and CSMAR database. It is obvious that open information channels ensure the compliance and scientificity of the research.

## 4. Empirical analysis

### 4.1 Variable calibration

Compared with traditional analysis methods, fsQCA method needs to calibrate the antecedent variables and outcome variables first, so as to convert the original data into 0–1 set data under Boolean algebraic logic, and assign set membership to the variables, that is, set the breakpoints. Preset three breakpoints: “fully in”, “crossover”, and “fully out”. The closer the value is to 1, the higher the membership degree is, and vice versa. On the basis of fully considering the distribution characteristics of sample data, the three breakpoints of the target set are set as the upper quartile (75%), the median and the lower quartile (25%). The calibration breakpoints and descriptive statistics of each variable are shown in Table 2. The calibration results are in Appendix 1 in S1 Appendix.

**Table 2:** Data calibration and descriptive statistical analysis of each variable.

Variable	Calibration			Descriptive statistics			
	Fully in	Crossover	Fully out	Mean	Std.Dev	Max	Min
OC	53.65	34.71	20.93	37.06	16.88	67.13	17.97
IB	40.59	37.50	33.33	34.94	10.09	50.00	9.09
EI	16.35	15.83	15.59	16.00	0.62	17.37	14.94
ASB	8	7	5	7.38	3.34	19	0
HHI	15.29	5.02	2.37	8.33	7.04	21.93	0.51
LQ	0.92	0.52	0.31	0.66	0.54	2.03	0.00
GC	18.17	16.64	15.26	16.58	1.72	19.18	12.88

### 4.2 Necessity test

In this paper, the necessity of single antecedent condition is tested before configuration analysis. It is generally believed that if the consistency is greater than or equal to 0.9, it meets the test standard of necessary conditions [88, 89], that is, the antecedent factor is a necessary condition for the outcome factor. By running the necessity condition test in fsQCA3.0 software, the results are shown in Table 3. The consistency level of the six antecedent variables is lower than 0.9, indicating that in the single antecedent conditions, the single condition itself is not a necessary condition for obtaining high-level green credit or low-level green credit. Therefore, the configuration analysis method is further used to study the combination of various conditional elements of green credit.

**Table 3:** Analysis of necessary conditions.

Condition tested	GC		~GC	
	Consistency	Coverage	Consistency	Coverage
OC	0.6565	0.6979	0.3440	0.3649
~OC	0.4026	0.3808	0.7152	0.6751
IB	0.4706	0.4875	0.6046	0.6249
~IB	0.6379	0.6178	0.5042	0.4872
EI	0.3821	0.3931	0.6643	0.6820
~EI	0.6909	0.6735	0.4088	0.6820
ASB	0.5130	0.4976	0.5859	0.5670
~ASB	0.5536	0.5726	0.4808	0.4963
HHI	0.8990	0.8988	0.2842	0.2835
~HHI	0.2833	0.2840	0.8985	0.8987
LQ	0.4046	0.4288	0.6393	0.6762
~LQ	0.6945	0.6587	0.4600	0.4353

The minimum consistency threshold is 0.75, which is generally set to 0.8 [90]. This paper sets the consistency threshold to 0.8 and the case frequency threshold to 1. When the case consistency threshold is higher than 0.8, the result variable is encoded as 1, and when it is lower than 0.8, the result variable is encoded as 0. At the same time, in order to reduce the potential contradictory configuration, the truth table row with PRI constant value less than 0.75 is excluded. Finally, it should note that 20 of the 72 sample cases passed the consistency threshold of high-level green credit. After constructing the truth table, the following empirical analysis is carried out. The truth table is shown in Appendix 2 in S1 Appendix.

### 4.3 Implementation path and case analysis

fsQCA method obtains three kinds of solutions: complex solutions (without using “logical remainder”), parsimonious solutions (using all “logical remainder”) and intermediate solutions (meaningful “logical remainder” is included in the solution). Because the complex solution does not simplify the configuration composition, it presents the most combination of factors. In each expression, there are core conditions and non-core conditions, which is an important feature to distinguish model paths. If the condition and result exist a strong causal relationship, the condition is the core condition; if the condition and result exist a weak causal relationship, the condition is non-core condition.

As shown in Table 4, five configuration paths of high-level green credit are obtained, of which the lowest consistency is 0.9126 and the highest is 0.9914, it indicates the sufficient conditions for generating high-level green credit in these five configurations. The consistency of the overall configuration is 0.9458, which shows that 94.58% of the banks show high-level green credit behavior in the cases that meet these five configurations. The coverage of the overall configuration is 0.5622, indicating that the five configurations jointly explain 56.22% of the cases. The consistency and coverage of the solutions in this paper are higher than the critical value, and it indicates that the empirical analysis is effective. Based on five conditional configuration paths, this paper further analyzes the differential adaptation relationship between the bank’s internal governance structure and external governance structure in improving the level of green credit. According to the core elements, the five configurations can be divided into two types. The consistency of the five configurations is 0.9914, 0.9126, 0.9467, 0.9843 and 0.9746 respectively, which all exceeded the minimum consistency threshold. In addition, the coverage of the five configurations are 0.4134, 0.2173, 0.1626, 0.3643 and 0.2980 respectively. According to the coverage, most banks obtain high-level green credit through configuration 1a, that is, configuration 1a is more important than other configurations.

**Table 4:** Configuration of high-level green credit.

Conditions		Solutions				
		1a	1b	1c	2a	2b
Internal Governance						
	OC	●	●	●	●	●
	IB		⊕	·		⊕
	EI	⊕	⊕		⊕	
	ASB		⊕	·	〇	〇
External Governance						
	HHI	·		·	●	●
	LQ	〇	〇	〇		
Consistency		0.9914	0.9126	0.9467	0.9843	0.9746
Raw coverage		0.4134	0.2173	0.1626	0.3643	0.2980
Unique coverage		0.0541	0.0033	0.0316	0.0297	0.0430
Solution coverage		0.5622				
Solution consistency		0.9458				

Note:●indicates that the exist of core causal conditions;·indicates that the exist of non core causal conditions;〇indicates the absence of core causal conditions;⊕indicates the absence of non core causal conditions; “Blank” indicates that the condition may or may not exist in the configuration [91].

Configurations 1a, 1b and 1c show that a combination of high shareholding ratio of the first largest shareholder and low non-performing asset ratio (good loan quality) can lead to high-level green credit. Obviously, there are 79.33% of banks have achieved a high-level green credit through such factors. Industrial and Commercial Bank of China, China Bohai bank, China Development Bank, China Construction Bank and China Everbright Bank are mainly in line with this configuration. Among them, 41.34% of banks also rely on the support of low executive compensation and weak market competition. The representative banks are China Development Bank and China Bohai bank. 21.73% of banks also rely on the support of weak independence of the Board, low executive compensation and low activity of Supervisory Board, such as China Development Bank and China Everbright Bank. Such a combination will produce a high-level green credit regardless of the degree of market competition. 16.26% of banks also relies on the support of strong independence of the Board, high activity of Supervisory Board and weak market competition. The representative banks are Industrial and Commercial Bank of China and China Construction Bank. Such a combination will produce a high-level green credit regardless of high or low executive compensation. From configurations 1a and 1b, there is a substitution between the poor independence of the Board and the low activity of Supervisory Board with low degree of market competition. From configurations 1a and 1c, there is a substitution between high independence of the Board and high activity of Supervisory Board with low executive compensation. Configuration 1b requires that all conditions do not exist except ownership concentration and degree of market competition (don’t care). Configuration 1C requires that all conditions exist except loan quality and executive incentive (don’t care).

Configurations 2a and 2b show that the combination of high shareholding ratio of the first largest shareholder, low degree of market competition and low activity of Supervisory Board can lead to high-level green credit. The existence of market competition as a necessary condition shows that external governance of Chinese Bank will also play an important role in green credit. There are 66.23% of banks have achieved a high-level green credit through such factors. Agricultural Bank of China, Industrial and Commercial Bank of China and China Development Bank are mainly in line with this configuration. Obviously, 36.43% of them also rely on the support of low executive compensation, and the representative banks are Agricultural Bank of China and Industrial and Commercial Bank of China; 29.8% also rely on the support of the weak independence of the Board, and the representative bank is China Development Bank. From these two configurations, there is a substitution between low executive compensation and weak independence of the Board.

At the same time, this paper lists the configurations that hinder the generation of high-level green credit, as shown in Table 5. There are 8 configurations, which can be roughly divided into 4 categories. The consistency of the overall configuration is 0.9510, which shows that 95.10% of the banks show low-level green credit behavior in the cases that satisfying these 8 configurations. The coverage of the overall configuration is 0.6635, indicating that these 8 configurations jointly explain 66.35% of the cases. The consistency of a single configuration is greater than 0.8, indicating that the influencing factors composed of all cases are sufficient conditions to hinder high-level green credit. Configurations 3a, 3b and 3c require a high degree of market competition. Configuration 4a requires that all conditions do not exist except for independence of the Board and loan quality (don’t care). Configuration 4b indicates that low ownership concentration, high degree of market competition and low loan quality will produce low-level green credit. Configurations 5a and 5b indicate that low ownership concentration, high degree of market competition and high executive compensation will produce low-level green credit. Configuration 6 indicates that low ownership concentration and high degree of market competition will produce low-level green credit. Finally, configuration 3b and 4b have the highest original coverage, indicating that most banks hinder high-level green credit through these two paths. Comparing the two configurations, it can be found that there is a substitution between low ownership concentration and high executive compensation.

**Table 5:** Configuration of hindering high-level green credit.

Conditions		Solutions							
		3a	3b	3c	4a	4b	5a	5b	6
Internal Governance									
	OC	·			〇	〇	〇	〇	〇
	IB		·			·	·	⊕	·
	EI	●	●	●	⊕		●	●	⊕
	ASB			·	⊕		⊕	·	·
External Governance									
	HHI	〇	〇	〇	〇	〇	〇	〇	〇
	LQ	●	●	●	●	●			
Consistency		0.9831	0.9875	0.9671	0.9788	0.9582	0.9960	0.9301	0.9205
Raw coverage		0.2108	0.3073	0.2778	0.1410	0.3056	0.1396	0.1849	0.1352
Unique coverage		0.0175	0.0047	0.0000	0.0373	0.0153	0.0384	0.0601	0.0072
solution coverage		0.6635							
solution consistency		0.9510							

Note:●indicates that the exist of core causal conditions;·indicates that the exist of non core causal conditions;〇indicates the absence of core causal conditions;⊕indicates the absence of non core causal conditions; “Blank” indicates that the condition may or may not exist in the configuration [91].

Through the above analysis, it can be found that the ownership concentration represented by the “shareholding proportion of the first largest shareholder” appears in all high-level green credit configurations, which has become an indispensable factor leading to the high-level green credit of banks. This shows the importance of the bank’s internal governance structure to green credit. At the same time, good loan quality and low degree of market competition are also important factors to produce high-level green credit. Thus, the impact of external governance structure of bank on green credit cannot be ignored. In order to improve the level of green credit, banks should pay attention to the combination of internal and external governance in the future to maximize its linkage effect. “HHI” appears in all low-level green credit configurations, and the high degree of market competition has become the most important condition for banks’ low-level green credit. Low ownership concentration, high executive compensation and high non-performing asset ratio will also generate low-level green credit. The configuration of high-level green credit and low-level green credit are not completely opposite, indicating that the configuration affecting green credit has causal asymmetry. In China, for the future development of banks, there is no need to pursue a completely unified governance model, the Chinese bank can establish an internal and external governance structure suitable for their own bank development according to the actual situation and improve the level of bank green credit.

### 4.4 Robustness test

In order to further verify the research results, this paper carries out a robustness test. Adjusting the consistency is an effective method to test the robustness of the empirical results in fsQCA [89]. Because the adjustment of consistency threshold will directly affect the number of configurations included in truth table analysis, and then affecting the results. In this paper, the consistency threshold is increased to 0.9, and the frequency remains unchanged. The sample data are reprocessed, and the data results are shown in Table 6. Compared with the previous results, the overall consistency increased from 0.9458 to 0.9744, and the overall coverage decreased from 0.5622 to 0.5586. Generally speaking, it is reasonable when the overall consistency is higher than 0.8 and the overall coverage is greater than 0.5, which is consistent with this study. The antecedent condition configurations are basically the same, and there are no conflicting results, indicating that the research conclusion of this paper is robust.

**Table 6:** Robustness test configuration table.

Conditions		Solutions			
		1	2a	2b	3
Internal Governance					
	OC	●	●	●	●
	IB			⊕	·
	EI	⊕	⊕		
	ASB		〇	〇	〇
External Governance					
	HHI	●	●	●	●
	LQ	〇			〇
Consistency		0.9920	0.9850	0.9755	0.9467
Raw coverage		0.4134	0.3643	0.2980	0.1625
Unique coverage		0.0541	0.0297	0.0430	0.0316
Solution coverage		0.5586			
Solution consistency		0.9744			

Note:●indicates that the exist of core causal conditions;·indicates that the exist of non-core causal conditions;〇indicates the absence of core causal conditions;⊕indicates the absence of non-core causal conditions; “Blank” indicates that the condition may or may not exist in the configuration [91].

## 5. Conclusions

How to optimize the bank governance structure and improve green credit is the focus of banks in China. fsQCA effectively identifies five configurations of high-level green credit of banks in China, indicating that the realization of high-level green credit by banks is not the result of a single factor, but the result of the joint action of relevant constituent factors of bank governance structure. These factors affect each other, interact and reasonably combine within a whole, so as to finally improve the level of green credit. Based on the case analysis of 12 banks from 2014 to 2019, this paper selects ownership concentration, independence of the Board, executive incentive, activity of Supervisory Board, degree of market competition and loan quality as the antecedent variables, and draws the following conclusions.

Firstly, among the five paths to achieve high-level green credit, the most covered sample banks are those with high ownership concentration and good loan quality. However, this does not mean that this governance structure can be applied to all banks and produce the same effect. Banks can choose the path to achieve high-level green credit or avoid low-level green credit by using the “overall perspective” and combining their existing conditions and characteristics. Secondly, the configuration of green credit has causal asymmetry. High ownership concentration is the key factor to produce high-level green credit, and high degree of market competition is the key factor to produce low-level green credit. Thirdly, in the internal governance structure, compared with the Board characteristics and the management status, there are more configurations with ownership concentration as the core factor, indicating that ownership structure occupies an essential position in China’s bank internal governance system and it is the most important factor affecting green credit. Fourth, there is a substitution between the low-level independence of the Board and the low-level executive compensation. The low activity of Supervisory Board and the low non-performing assets ratio are also substitutable to a certain extent.

The theoretical contribution of this paper mainly includes the following three aspects. Firstly, combined with the actual situation of Chinese banks, starting from the internal governance structure and external governance structure, this paper puts forward an integrated analysis framework of “internal governance structure- external governance structure- green credit”. Secondly, based on the “configuration perspective”, this paper uses fsQCA method to find the diversity of configurations affecting green credit and the interdependence of various factors in the configuration. It not only reveals the five modes affecting green credit, but also clarifies the most effective methods to improve the level of green credit, which is conducive to an in-depth explanation of green credit, a complex phenomenon affected by multiple factors. Moreover, this paper finds that the ownership concentration, independence of the Board, executive incentive, activity of Supervisory Board, degree of market competition and loan quality cannot solely constitute the essential conditions for high-level green credit or low-level green credit, indicating that the six conditions of internal governance structure and external governance structure work together on green credit in linkage and matching. Finally, this paper finds that the configuration affecting green credit has causal asymmetry, which makes up for the defect that the traditional regression research cannot explain the literature of high-level green credit, which is difficult to be applied to the management practice of low-level green credit. The path of generating high-level green credit is not completely opposite to the path of hindering high-level green credit.

Theoretical analysis and empirical test results show that bank governance structure has an important impact on green credit, and reasonable matching and linkage can help us to achieve a high level of green credit. The method of this paper is different from the traditional quantitative research. Although the conclusion of this method has made a certain contribution to the research field of green performance of bank in China, there are still deficiencies in some aspects. Firstly, the cases collected in this paper are limited and do not cover all possible paths. Secondly, the bank governance structure is a very complex system, which has many influencing factors, and the configuration effect between factors is ever-changing. However, limited to the availability of research data, in order to prevent the emergence of limited diversity, the antecedent variable in this paper only selects the most representative six indicators, and does not include all the influencing factors of internal governance and external governance. In future research, it will collect more cases and introduce more factors for further discussion.

## Supporting information

S1 File (XLSX)

S1 Appendix (DOCX)
