# Supplementary material for: The influence of bank governance structure on green credit
Source: PLoS One. 2023 Mar 13;18(3):e0281115. doi: 10.1371/journal.pone.0281115 (PMC10010550; doi:10.1371/journal.pone.0281115)
Supplement: S1 Appendix — (DOCX) [file pone.0281115.s002.docx]

# Appendix

**Appendix 1.** Calibration results

|  | OC | IB | EI | ASB | HHI | LQ | GC |
| --- | --- | --- | --- | --- | --- | --- | --- |
| case1 | 0.92 | 0.00 | 0.63 | 0.50 | 0.67 | 0.00 | 0.99 |
| case2 | 0.57 | 0.00 | 0.01 | 0.05 | 0.68 | 0.00 | 0.99 |
| case3 | 0.57 | 0.00 | 0.20 | 0.00 | 0.63 | 0.00 | 0.99 |
| case4 | 0.57 | 0.00 | 0.10 | 0.00 | 0.49 | 0.02 | 0.99 |
| case5 | 0.57 | 0.00 | 0.00 | 0.00 | 0.63 | 0.00 | 0.99 |
| case6 | 0.57 | 0.00 | 0.47 | 0.00 | 0.55 | 0.09 | 0.99 |
| case7 | 0.99 | 0.22 | 0.98 | 0.05 | 0.95 | 0.04 | 0.76 |
| case8 | 0.99 | 0.22 | 0.53 | 0.01 | 0.94 | 0.41 | 0.85 |
| case9 | 0.99 | 0.01 | 0.10 | 0.05 | 0.95 | 0.51 | 0.88 |
| case10 | 0.99 | 0.01 | 0.52 | 0.18 | 0.94 | 0.72 | 0.91 |
| case11 | 0.99 | 1.00 | 0.24 | 0.01 | 0.93 | 0.84 | 0.93 |
| case12 | 0.99 | 0.31 | 0.51 | 0.95 | 0.93 | 0.61 | 0.95 |
| case13 | 0.97 | 0.99 | 0.87 | 0.50 | 0.98 | 0.01 | 0.89 |
| case14 | 0.97 | 0.50 | 0.25 | 0.95 | 0.98 | 0.06 | 0.95 |
| case15 | 0.97 | 1.00 | 0.31 | 0.95 | 0.98 | 0.03 | 0.96 |
| case16 | 0.97 | 0.05 | 0.47 | 0.95 | 0.98 | 0.03 | 0.97 |
| case17 | 0.97 | 0.99 | 0.14 | 0.18 | 0.98 | 0.06 | 0.97 |
| case18 | 0.97 | 0.22 | 0.07 | 0.18 | 0.98 | 0.07 | 0.98 |
| case19 | 0.52 | 1.00 | 0.86 | 0.95 | 0.99 | 0.08 | 0.96 |
| case20 | 0.50 | 0.05 | 0.00 | 0.95 | 0.99 | 0.45 | 0.96 |
| case21 | 0.50 | 0.22 | 0.00 | 0.50 | 0.99 | 0.59 | 0.97 |
| case22 | 0.50 | 0.50 | 0.14 | 0.95 | 0.99 | 0.45 | 0.98 |
| case23 | 0.50 | 1.00 | 0.02 | 0.50 | 0.99 | 0.00 | 0.98 |
| case24 | 0.50 | 0.99 | 0.00 | 0.05 | 0.99 | 0.62 | 0.98 |
| case25 | 0.71 | 0.01 | 0.43 | 0.50 | 0.97 | 0.09 | 0.88 |
| case26 | 0.70 | 0.22 | 0.00 | 1.00 | 0.96 | 0.31 | 0.91 |
| case27 | 0.70 | 0.22 | 0.01 | 0.05 | 0.96 | 0.92 | 0.93 |
| case28 | 0.70 | 0.05 | 0.01 | 0.18 | 0.96 | 0.00 | 0.95 |
| case29 | 0.70 | 0.98 | 0.00 | 0.18 | 0.97 | 0.57 | 0.97 |
| case30 | 0.70 | 0.72 | 0.10 | 0.05 | 0.96 | 0.13 | 0.98 |
| case31 | 0.04 | 0.92 | 0.00 | 0.50 | 0.02 | 0.93 | 0.00 |
| case32 | 0.04 | 1.00 | 0.14 | 0.01 | 0.02 | 1.00 | 0.00 |
| case33 | 0.04 | 0.72 | 0.00 | 0.95 | 0.02 | 1.00 | 0.00 |
| case34 | 0.81 | 0.92 | 0.81 | 0.18 | 0.02 | 1.00 | 0.00 |
| case35 | 0.81 | 0.99 | 0.99 | 0.05 | 0.02 | 1.00 | 0.01 |
| case36 | 0.81 | 0.05 | 0.99 | 0.18 | 0.03 | 1.00 | 0.01 |
| case37 | 0.04 | 0.17 | 0.98 | 1.00 | 0.25 | 0.10 | 0.46 |
| case38 | 0.04 | 0.99 | 0.00 | 1.00 | 0.35 | 0.26 | 0.51 |
| case39 | 0.04 | 0.99 | 0.70 | 1.00 | 0.48 | 0.56 | 0.52 |
| case40 | 0.05 | 1.00 | 0.15 | 1.00 | 0.50 | 1.00 | 0.53 |
| case41 | 0.05 | 0.92 | 0.00 | 1.00 | 0.41 | 1.00 | 0.62 |
| case42 | 0.05 | 0.22 | 0.71 | 1.00 | 0.44 | 1.00 | 0.64 |
| case43 | 0.03 | 0.05 | 1.00 | 1.00 | 0.53 | 0.63 | 0.44 |
| case44 | 0.03 | 0.50 | 1.00 | 1.00 | 0.58 | 1.00 | 0.46 |
| case45 | 0.03 | 0.05 | 1.00 | 0.18 | 0.60 | 0.99 | 0.41 |
| case46 | 0.03 | 0.50 | 1.00 | 0.95 | 0.62 | 0.77 | 0.46 |
| case47 | 0.03 | 0.50 | 1.00 | 0.50 | 0.63 | 0.75 | 0.49 |
| case48 | 0.03 | 0.17 | 1.00 | 1.00 | 0.63 | 0.81 | 0.52 |
| case49 | 0.99 | 0.31 | 1.00 | 1.00 | 0.26 | 0.52 | 0.02 |
| case50 | 0.99 | 0.50 | 1.00 | 1.00 | 0.34 | 0.98 | 0.04 |
| case51 | 0.99 | 1.00 | 0.98 | 0.95 | 0.42 | 0.98 | 0.06 |
| case52 | 0.99 | 1.00 | 1.00 | 1.00 | 0.42 | 0.99 | 0.10 |
| case53 | 0.99 | 0.92 | 1.00 | 1.00 | 0.36 | 1.00 | 0.11 |
| case54 | 0.99 | 0.92 | 1.00 | 1.00 | 0.43 | 1.00 | 0.11 |
| case55 | 0.74 | 0.05 | 0.90 | 0.50 | 0.06 | 0.05 | 0.03 |
| case56 | 0.09 | 0.50 | 0.84 | 0.50 | 0.08 | 0.10 | 0.03 |
| case57 | 0.11 | 0.22 | 0.84 | 0.50 | 0.08 | 0.05 | 0.08 |
| case58 | 0.12 | 0.99 | 0.83 | 0.05 | 0.07 | 0.04 | 0.15 |
| case59 | 0.12 | 1.00 | 0.83 | 0.18 | 0.10 | 0.98 | 0.16 |
| case60 | 0.12 | 0.92 | 1.00 | 0.95 | 0.14 | 0.97 | 0.21 |
| case61 | 0.04 | 0.79 | 1.00 | 0.50 | 0.03 | 0.16 | 0.04 |
| case62 | 0.04 | 0.50 | 0.06 | 0.05 | 0.03 | 0.38 | 0.04 |
| case63 | 0.04 | 0.92 | 0.00 | 0.05 | 0.03 | 0.71 | 0.05 |
| case64 | 0.04 | 0.05 | 0.01 | 0.01 | 0.03 | 0.79 | 0.08 |
| case65 | 0.04 | 0.17 | 0.20 | 0.05 | 0.03 | 0.77 | 0.09 |
| case66 | 0.04 | 0.92 | 0.82 | 1.00 | 0.04 | 1.00 | 0.16 |
| case67 | 0.11 | 0.00 | 0.00 | 0.50 | 0.01 | 0.00 | 0.00 |
| case68 | 0.11 | 0.00 | 0.03 | 0.50 | 0.01 | 0.00 | 0.00 |
| case69 | 0.11 | 0.00 | 0.20 | 0.50 | 0.01 | 0.00 | 0.01 |
| case70 | 0.11 | 0.00 | 0.54 | 0.50 | 0.01 | 0.00 | 0.01 |
| case71 | 0.11 | 0.00 | 0.70 | 0.50 | 0.01 | 0.02 | 0.01 |
| case72 | 0.11 | 0.97 | 0.81 | 0.50 | 0.01 | 0.00 | 0.01 |

**Appendix 2.** The truth table of high-level green credit

| OC | IB | EI | ASB | HHI | LQ | number | GC | Raw consist. |
| --- | --- | --- | --- | --- | --- | --- | --- | --- |
| 1 | 0 | 0 | 0 | 1 | 0 | 6 | 1 | 0.9847 |
| 1 | 1 | 0 | 0 | 1 | 0 | 2 | 1 | 0.9812 |
| 1 | 1 | 0 | 1 | 1 | 0 | 1 | 1 | 0.9806 |
| 1 | 0 | 0 | 1 | 1 | 0 | 2 | 1 | 0.9788 |
| 1 | 0 | 1 | 0 | 1 | 0 | 2 | 1 | 0.9744 |
| 1 | 0 | 0 | 0 | 1 | 1 | 2 | 1 | 0.9649 |
| 1 | 1 | 0 | 0 | 1 | 1 | 2 | 1 | 0.9592 |
| 1 | 0 | 1 | 0 | 1 | 1 | 1 | 1 | 0.9488 |
| 1 | 1 | 1 | 1 | 1 | 0 | 1 | 1 | 0.9094 |
| 1 | 0 | 0 | 0 | 0 | 0 | 1 | 1 | 0.8136 |
